# A 30-year journey in wildlife assisted reproductive technologies

**DOI:** 10.1590/1984-3143-AR2026-0101

**Published:** 2026-09-21

**Authors:** Pierre Comizzoli

**Affiliations:** 1 Department of Reproductive Sciences, Smithsonian’s National Zoo and Conservation Biology Institute, Washington, DC, United States of America

**Keywords:** wildlife, fertility, conservation biology, assisted reproduction, biobanking

## Abstract

Assisted reproductive technologies (ARTs) have been extensively developed in wild animal species over the last thirty years to become effective instruments for biodiversity conservation. These technologies, which were first adapted from advances in livestock species management and human medicine, have developed into more specialized methods suited to the particular anatomical and physiological traits of wild animal species. This article examines the development, improvement, and use of ARTs for animal conservation throughout a thirty-year period, emphasizing significant turning points, enduring difficulties, and potential future paths.

## Introduction

The accelerating loss of biodiversity has driven our scientific community to explore innovative conservation strategies. Among these, assisted reproductive technologies (ARTs) have gained prominence as complementary tools to conservation breeding (Mastromonaco, 2024). These cutting-edge biotechnologies can help to propagate small animal populations while overcoming infertility issues. Over the past 30 years, efforts have focused on understanding reproductive biology across diverse taxa, translating that knowledge into practical interventions as well as developing ARTs.

As experts in wildlife reproduction, we have constantly challenged conventional dogma coming from studies conducted in laboratory species or livestock. Our findings have created added information helpful to sustain populations of threatened wildlife, ranging from coral reefs to megafauna. The objective of this review is to (1) look back at the past 30 years of research and accomplishments in wildlife ART and (2) foresee what will happen in the coming decade.

## The 1990s – Adapting domestic models to our needs

The early phase of wildlife ART development relied heavily on protocols established for livestock and humans. Techniques such as artificial insemination (AI), in vitro fertilization (IVF), and embryo transfer were tested in a limited number of wild species. However, significant interspecies differences in reproductive physiology quickly became apparent (Loskutoff et al., 1995). These discrepancies underscored the necessity of species-specific research rather than direct extrapolations from companion or domestic animals. During the 1990s, foundational work in non-invasive endocrine monitoring, gamete collection, and cryopreservation also laid the groundwork for future advancements. Importantly, the concept of genome resource banking (GRB) began to take shape, emphasizing the need for long-term preservation of genetic diversity in organized biorepositories (Wildt et al., 1997).

The first wild species that I personally studied were the collared peccary and the white-lipped peccary in French Guyana. This first exposure to wildlife science was very inspiring as I learned the surprising traits of reproduction in Tayassuids that are so different from the domestic pig (Silva et al., 2023). I then investigated new mechanisms in early embryo development in vitro using alternate animal models for wildlife, such as bovine and deer species. We found that there was a strong paternal effect at the beginning of the embryo development that determined the success of pregnancy and birth of healthy offspring (Comizzoli et al., 2000, 2001, 2003). I then had the opportunity to confirm a similar paternal effect in early embryo from domestic cats (Comizzoli et al., 2006). By providing evidence and simple approaches, this body of work contributed to new standards of genitor selection in assisted reproductive programs to sustain rare genotypes and endangered species.

In the 1990s, successful births of wild ungulates and carnivores were obtained through conventional ART even though the success rate remained way lower than in domestic species (Pukazhenthi and Wildt, 2004). However, the first cloned animals produced by transferring a somatic cell to an enucleated oocyte, starting with Dolly the sheep and quickly followed by other livestock species, thrilled the conservation community in the late 1990s (Campbell et al., 1996; Polejaeva et al., 2000). It was rapidly assumed that the same techniques could be applied to all wild creatures and that the technology would eventually aid in the conservation of several animal species. In order to create cloned embryos, an astounding number of research teams transferred the nuclei of cells from a variety of wild species (from giant pandas to endangered antelopes) into enucleated oocytes from common species (mice, rabbits, cats, and cattle, to name a few) which were believed to be universal recipients for reprogramming cell nuclei from any species (Dominko et al., 1999; Cowl et al., 2023). All reports and scientific studies in various species at that time clearly indicated that a large number of cells and oocytes were needed to effectively develop only a few growing embryos that might be transplanted to surrogate females. Most research projects stopped at the embryo production stage because transplanting embryos was (and still is) expensive, especially in large animal species. Due to the use of enucleated oocytes from species that were different from the donor cell nucleus, the genetic ‘authenticity’ of the cloned embryos was also contested (Adams et al., 2024). Indeed, the recipient oocyte also carries some genetic information through mitochondria even if the original nucleus is removed.

Retrospectively, this body of research emphasized how important it was to study reproductive biology more thoroughly and develop ARTs in species that are still abundant. Unfortunately, advanced tools like cloning could not be improved fast enough to address extreme situations when there were not enough individuals left from an almost extinct species to study.

## The 2000s – Going back to the basics and expanding the horizons

The low success of ARTs in wildlife reminded our scientific community that we still had a lot to learn about the principles of reproductive biology across a wide range of animals (Mastromonaco and Comizzoli, 2018). It is a necessary precondition if we want to progress in any area, including the development of assisted reproduction. It already took a long time to optimize behavioral and physiological tests to generate accurate data, including with non-invasive techniques (e.g., monitoring testicular or ovarian activity through sexual hormone metabolites in the urine or in feces). Additionally, neither the general public nor potential investors were attracted to ‘conventional’ approach that were not necessarily spectacular like the birth of a cloned animal. We still have today to address the same issue by further communicating and raising awareness about the values of fundamental reproductive biology.

The beginning of the 21^st^ century was a period of fast expansion. Sperm and embryos from a growing variety of species were successfully frozen and thawed thanks to advances in comparative cryobiology (Pukazhenthi et al., 2006). Interdisciplinary partnerships also became more robust during that time. To include ARTs into more comprehensive conservation frameworks, reproductive biologists, veterinarians, ecologists, and geneticists increased their collaborations. Notable achievements were reported in ungulates (Morrow et al., 2009; Thongphakdee et al., 2017) and included impressive results in the restoration of populations of black-footed ferrets (Howard et al., 2016) or giant pandas (Martin-Wintle et al., 2019). In addition to those contributions, our group started to document cryopreservation of oocytes at the germinal vesicle stage in the domestic cat as a model for wild felids (Comizzoli et al., 2004, 2008, 2009, 2011). During that same decade, the scope of ARTs was also expanded by innovative approaches including testicular and ovarian tissue cryopreservation in wild species, with the idea of subsequently producing mature gametes in vitro or by xenografting (Comizzoli et al., 2012).

## The past decade – Integrating innovations into conservation actions

Among new techniques that have surfaced in different wild species, the oviductal artificial insemination in wild felids is a good example of an approach that has had an impact on wildlife ART. Cat semen was traditionally deposited directly into the uterus by laparoscopy. Sometimes there were not enough sperm cells, either in terms of quantity or quality, to ensure the success of the procedure. A group of colleagues came up with the idea of putting a small amount of semen in the oviduct, closer to the eggs, with more chances to fertilize them (Swanson, 2023). This modification to the insemination process has produced several kittens from a variety of cat species, including the ocelot and the clouded leopard. The translation and use of the oviductal technique was then successfully implemented for early embryo transfers in cheetahs (Crosier et al., 2020).

Another technology that shifted the paradigm of wildlife conservation and reproduction management was the high-throughput DNA sequencing, which is the ability to read a species' entire genetic code much faster and more precisely. DNA sequencing has already profoundly changed how the human genome is described and understood while opening up numerous new possibilities for the characterization of diseases and the creation of treatments (Collins and Talkowski, 2025). Concurrently, conservation biology has integrated the genomics revolution to improve taxonomy, individual and population genetic architecture, and genetic management (Wildt et al., 2019). Further techniques have also been developed to fully characterize how DNA is transcribed into RNAs (transcriptomics), how those RNAs are translated into proteins (proteomics), and how proteins and other elements interact in vital activities (metabolomics), including reproduction (Holt and Comizzoli, 2026). Knowing how to successfully integrate those new omics tools into animal protection, especially in the reproductive sciences, is key. Meanwhile, these approaches are generating enormous volumes of data that might quickly become overwhelming; this has to be addressed as well. More recently, genomic technologies have also significantly enhanced the detection and characterization of naturally occurring microbial communities on animal bodies. The concept of reproductive microbiomes is now growing as new dimension in conservation biology (Comizzoli et al., 2021).

We would be remiss if we were not talking about stem cells. Stem cell research has made a lot of progress in wildlife conservation with more potential applications to preserve species fertility and save them from extinction (Hutchinson et al., 2024). In parallel, wildlife GRBs have evolved to include induced pluripotent stem cells.

Lastly, in the most recent years, our group has been focused on the exploration of alternate preservation methods for germplasms using the domestic cat model. These studies demonstrate the possibility of storing nuclei as well as immature oocytes at ambient temperatures (Elliott et al., 2015; Lee and Comizzoli, 2019; Lee et al., 2019, 2022, 2023; Amelkina and Comizzoli, 2020).

## Future directions

Looking ahead, there are several priorities and promising avenues. Pursuing the integration data from genomic and related omics tools data to guide ART applications is needed. Emerging technologies such as genome editing and synthetic biology may also play crucial roles, though they require careful regulation and ethical scrutiny.

Crucially, we must link the vast quantity of available data about the phenotypes or visible traits of wild species with databases created by the omics methods (from genomes to microbiomes). Indeed, physical attributes, physiological qualities, habits, and disease susceptibility are all included in what is now known as the phenome. Because genetic or microbiome differences, environmental influences, and molecular processes combine to form an organism's features, studying the link between the genome and the phenome is essential in wildlife reproduction and ART. In that area, big data management, machine learning, and artificial intelligence are already becoming crucial for gathering, analyzing, and interpreting data.

Improving viability and developmental competence of oocytes from wild species after cryopreservation remains a persisting challenge that we need to tackle. A related priority is about the expansion of wildlife biobanking while enhancing global collaborations and standardization of preservation protocols, including the development of tools suitable for field use.

## Conclusion – Challenges and lessons learned

The development of wildlife ARTs over the past 30 years illustrates both scientific creativity and the pressing need to conserve biodiversity. ARTs are becoming essential instruments in the conservation toolbox, despite ongoing difficulties. Each species presents unique reproductive traits, requiring tailored approaches. Many endangered species remain poorly studied. Field conditions often complicate sample collection and technology deployment.

Notwithstanding the initial excitement surrounding a technology like cloning to rescue species from extinction, this approach has been tempered by practical constraints and ethical issues. Although cutting-edge reproductive technologies can aid in conservation, they cannot stop the decline in wildlife populations on their own. Rather, interdisciplinary methods that incorporate reproductive biology, environmental science, and ethical dimensions are crucial. Given how environmental stressors like contaminants (including endocrine disruptors and microplastics) and climate change can impact reproductive success across generations, it also is crucial to comprehend the developmental roots of health and disease in wildlife.

Lastly, in order to guarantee that wildlife ARTs are successful and sustainable in the face of persistent environmental and human-caused difficulties, we need to improve interdisciplinary collaborations, training programs, capacity building in biodiversity-rich regions, and public awareness.

## Data Availability

No research was used.
